# Knowledge Landscape and Hotspots of Research in Transthyretin Amyloid Cardiomyopathy: A Bibliometric Analysis

**DOI:** 10.7150/ijms.101888

**Published:** 2025-02-28

**Authors:** Yanzhi Liu, Xinqing Li, Anran Xin, Yuhui Zhang, Jian Zhang

**Affiliations:** 1 Heart Failure Center, State Key Laboratory of Cardiovascular Disease, National Center for Cardiovascular Diseases, Fuwai Hospital, Chinese Academy of Medical Sciences and Peking Union Medical College, Beijing, 100037, China.; 2 Key Laboratory of Clinical Research for Cardiovascular Medications, National Health Committee, Beijing, 100037, China.

**Keywords:** transthyretin amyloid cardiomyopathy, heart failure, tafamidis, molecular therapeutics, bibliometric analysis

## Abstract

**Background:** Transthyretin amyloid cardiomyopathy (ATTR-CM) is a progressive and frequently underdiagnosed cause of heart failure characterized by the pathological deposition of misfolded transthyretin (TTR) proteins in the cardiac tissue, leading to a poor prognosis and a significant reduction in quality of life. Despite its severity, therapeutic options remain limited, and knowledge gaps persist. This study aims to construct a knowledge map and identify research hotspots within the field of ATTR-CM.

**Methods:** Data were extracted from the Web of Science Core Collection (WoSCC), covering the period from January 1, 2000 to June 1, 2024. Bibliometric analyses were supplemented by qualitative assessments. VOSviewer, CiteSpace, and Bibliometrix were used to visualize academic community clusters, collaboration and citation networks to identify trends and hotspots in ATTR-CM research.

**Results:** A total of 1855 publications were analyzed. Contributions from multiple disciplines fueled a consistent upward trend in publications and citations. Europe and the United States dominated ATTR-CM research, with Mathew S. Maurer as the most prolific author, and the University of London as the leading research institution. The journals publishing these documents and references demonstrated credibility and broad disciplinary coverage. Reference analysis identified 10 main research fields. Keyword analysis unveiled five promising themes for research: early diagnosis and prognosis algorithm, specific medication development, management of comorbidities and complications, epidemiology and genotype-phenotype correlation, and molecular biology and mechanisms.

**Conclusion:** This study is the first comprehensive bibliometric analysis of the ATTR-CM field, supplemented by qualitative assessments. It systematically examines development trends, academic networks, and research themes, while identifying research hotspots and proposing future directions and approaches. These findings provide valuable insights to deepen the understanding of ATTR-CM and may foster advancements in scientific research and clinical applications.

## Introduction

Heart failure is a major global public health concern and a leading cause of hospitalization and mortality 1. Transthyretin amyloid cardiomyopathy (ATTR-CM), also referred to as transthyretin cardiac amyloidosis (ATTR-CA), is a frequently under-recognized cause of heart failure associated with a poor prognosis, particularly among the elderly population 2. Previous studies demonstrated that approximately 13% of elderly patients with heart failure with preserved ejection fraction (HFpEF) were affected by ATTR-CM 3, and a substantial proportion of ATTR-CM patients also present with heart failure with reduced ejection fraction (HFrEF) and right heart failure 4. However, the median survival after diagnosis in inadequately treated ATTR-CM patients ranges from 31 to 69 months 5. The delayed or missed diagnosis of -CM is primarily attributed to its nonspecific clinical presentation, which closely resembles hypertensive heart disease or hypertrophic cardiomyopathy, insufficient clinician awareness, and the dependence on specialized diagnostic tools including endomyocardial biopsy.

Amyloidosis is a group of disorders characterized by the extracellular deposition of misfolded protein aggregates 6. ATTR-CM occurs when misfolded transthyretin (TTR) protein accumulates in the heart, leading to ventricular wall thickening and impaired cardiac function. ATTR-CM is the cardiac manifestation of transthyretin amyloidosis (ATTR amyloidosis), one of the most clinically significant forms of amyloidosis. Depending on the presence or absence of TTR gene mutations, ATTR is classified into two subtypes: variant ATTR amyloidosis (ATTRv amyloidosis) or wild-type ATTR amyloidosis (ATTRwt amyloidosis) 7. Beyond cardiac involvement, ATTR amyloidosis can involve multiple organ systems, such as the gastrointestinal and nervous systems, resulting in a wide spectrum of clinical manifestations 8. This underscores the need for multidisciplinary care and comprehensive research to optimize the management of patients with ATTR-CM. While liver transplantation and advancements in TTR-targeted therapies have improved outcomes for certain patients, therapeutic options for ATTR-CM remain scarce, and substantial gaps in the understanding of its pathophysiology and management persist 9, 10. Hence, ATTR-CM research is essential to improving patient prognosis and developing targeted therapies.

Bibliometrics provides a systematic approach to mapping the knowledge landscape and identifying research hotspots by analyzing large volumes of literature. Using this methodology, this study aims to analyze the current literature on ATTR-CM with the following objectives: (1) to identify seminal studies, key contributors, and academic community networks, (2) to uncover research trajectories and thematic developments in ATTR-CM, (3) to highlight underexplored areas, providing a foundation for further investigation and guiding future research directions to drive advancements in the field of ATTR-CM. Considering that citation-based metrics may not fully capture the full multidimensional value of a publication or research entity, bibliometric analyses were supplemented with qualitative assessments to ensure a more comprehensive evaluation.

## Methods and materials

### Database selection and search strategies

Data were downloaded from the Web of Science Core Collection (WoSCC, Clarivate Analytics, Philadelphia, USA). To ensure consistency and reproducibility, the search was performed on July 17, 2024. The following search query were used: TS = (transthyretin amyloid cardiomyopathy) OR (transthyretin cardiomyopathy) OR (ATTR-CM) OR (transthyretin cardiac amyloidosis) OR (ATTR-CA) OR (ATTR amyloidosis-related cardiomyopathy) OR (ATTR cardiomyopathy).

### Inclusion and exclusion criteria

The criteria are as follows: (1) Time span**:** Since the early 21st century, cardiac amyloidosis, particularly ATTR-CM, has been increasingly recognized as an important etiology of cardiomyopathy. Therefore, literature published between January 1, 2000 and June 1, 2024 was included. (2) Language selection: Given that English is the primary language of international academic communication, only English-language publications were included to ensure comparability. (3) Publication types**:** The focus was restricted to original articles and reviews, as these publication types are systematically structured, ensuring academic rigor and providing clear insights into the research frontiers. To maintain consistency and relevance, meeting abstracts, case reports, editorials, letters, and patents were excluded. (4) Exclusion of duplicate records: Duplicate records were identified and removed.

### Extraction of bibliometric data

Two reviewers (LXQ and XAR) extracted the bibliometric data from each article. In case of disagreement between reviewers, consensus was reached through discussion or resolved by the senior author (ZYH). Bibliometric data were obtained by downloading the "plain text" version of the full record and cited references from WoSCC.

### Analysis and visualization

#### Quantitative metrics and qualitative evaluation tools

Classical bibliometric quantitative metrics including H-index, Number of Publications (NP), Average Citation per Publication (ACP), and Number of Citations (NC) were obtained from WoSCC. Journal information, including Impact Factor (IF), Journal Citation Reports (JCR) categories, Article Influence Score (AIS), and Percentage of Citable Open Access (PCOA), was obtained from https://jcr.clarivate.com to assess the quality of scientific information from various aspects. The fractional individualized H-index for journals was calculated using Publish or Perish (https://harzing.com/resources/publish-or-perish) to address the limitations of the standard H-index. The Altmetric Attention Score (AAS), sourced from Altmetric (https://www.altmetric.com), measures the visibility and impact of publications across non-traditional academic platforms. The F1000 Article Factor (FFa), derived from weighted expert evaluations via Faculty Opinions (https://connect.h1.co), provides a qualitative measure of academic impact.

#### Bibliometric tools

Microsoft Excel 2019 (Redmond, Washington) was used to process data by calculating the total growth rate, drawing a histogram, line charts, and a doughnut plot, plotting the growth curve of publications and creating three-line tables for data presentation.

VOSviewer (version 1.6.20, Centre for Science and Technology Studies, Leiden University, Nees Jan van Eck and Ludo Waltman, Leiden, the Netherlands) was used to visualize academic communities and collaboration networks among research entities, including authors, institutions, and countries 11. The co-authorship analysis module was applied, with nodes representing research entities and links indicating collaboration relationships. Link thickness reflects the frequency of cooperation, while the node and label size are proportional to total link strength (TLS). TLS represents the total strength of all connections for an entity, with higher TLS indicating greater connectivity or influence within the network. Academic communities are represented by different colors.

CiteSpace (version 6.3.R1 advanced, Drexel University, Chaomei Chen, Philadelphia, USA) was used to visualize citation networks over time through segmented time slices, which are represented by citation annual rings. In this study, the overlay maps module and analysis module for cited authors, references, and keywords were applied to (1) identify and remove duplicate publications, (2) construct co-citation networks of authors and references, dual-maps of citing and cited journals, and keyword co-occurrence networks, (3) visualize reference clusters as timeline maps 12, and (4) analyze citation bursts of highly cited references and generate a burst list 13. Each citation tree-ring node represents an element (cited author, reference, or keyword) with ring colors corresponding to the citation year. The ring thickness reflects the citation frequency in a specific year, and the overall node size corresponds to total citation frequency. Links between nodes represent co-citation relationships. Centrality measures the importance of an element within the research network. Nodes with a centrality value of 0.1 or greater are identified as critical bridges within the co-citation network. These nodes are highlighted by purple rings, with label size proportional to centrality. Citation bursts signify a sudden increase in citations for a reference within a short period. High burst strength is indicated by red segments within the nodes.

Bibliometrix (R version 4.4.0, University of Naples Federico II, K-Synth Srl, Italy), an open-source R tool for bibliometric analysis, is effective in identifying thematic classifications and trends within research fields through keyword analysis 14. In this study, the Thematic Evolution and Thematic Map tools in its Network Approach module were used to construct a thematic evolution map across research periods and construct a two-dimensional thematic plot based on keyword analysis. Additionally, Bibliometrix was used to identify core authors and journals using Lotka's Law and Bradford's Law.

## Results

### General data

**Figure 1:**
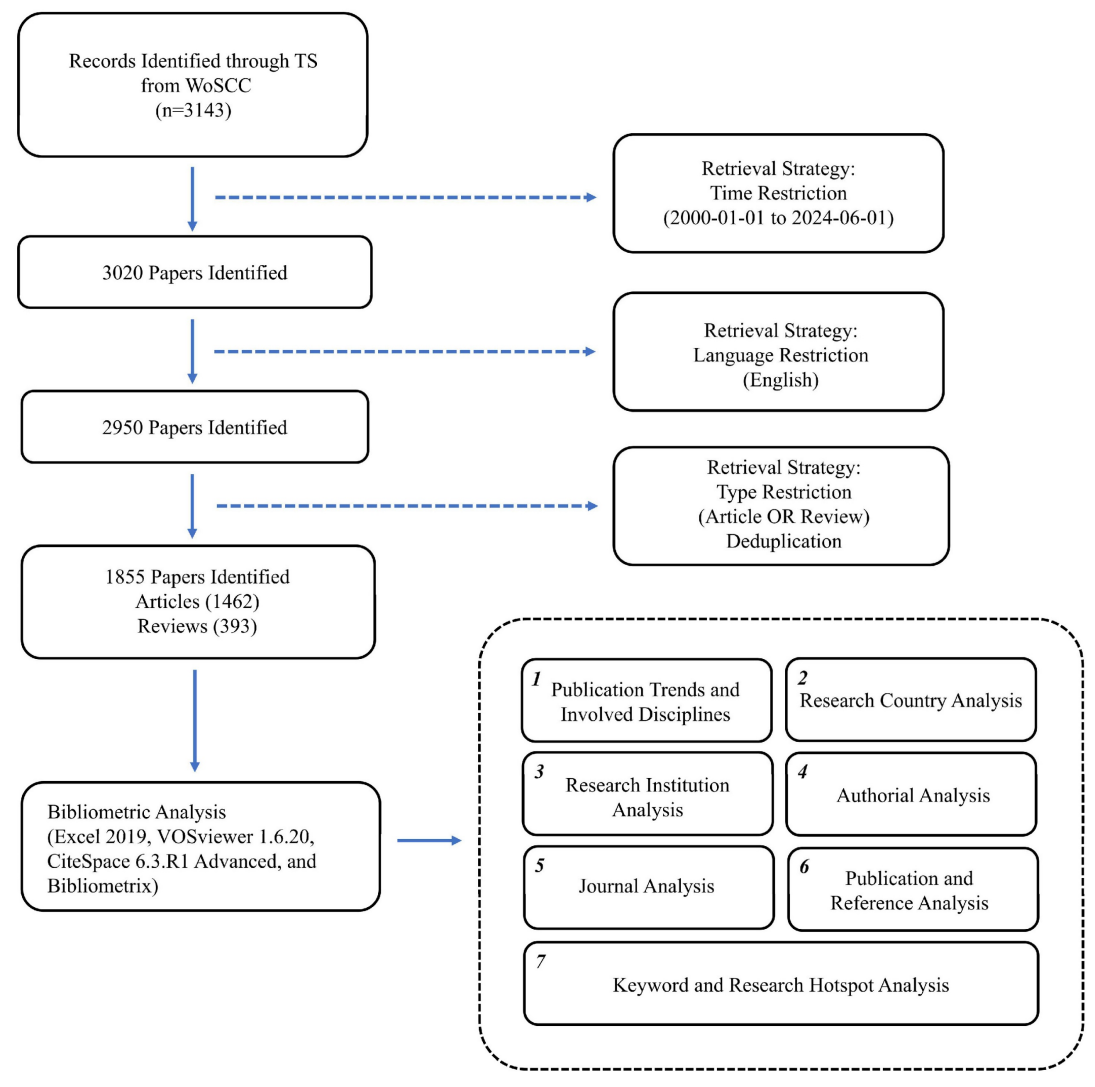
Study flowchart. Abbreviations: TS, topic search; WoSCC, Web of Science Core Collection.

A total of 3143 literature records were initially retrieved. After limiting the time span from January 1, 2000, to June 1, 2024 (123 excluded), confining to English-language publications (70 excluded), restricting to original articles and reviews (1095 excluded), and removing duplicates (0 excluded), 1855 records were included and analyzed, comprising 1462 articles and 393 reviews. To further explore the knowledge foundation, all 22872 references of these 1855 publications were also analyzed. The specific steps are illustrated in Figure 1.

### Publication trends and involved disciplines

**Figure 2:**
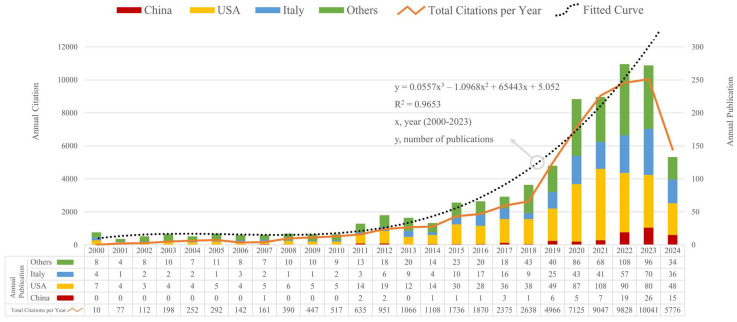
Trends in publications and citations for ATTR-CM. Stacked bars represent annual publications by country. Line chart shows total annual citations, and dotted line indicates the publication trend.

Over the past 24 years, the global annual number of publications and citations concerning ATTR-CM has risen steadily. The simulation of time series data indicates that the growth pattern of the global annual publication counts closely resembles an upward polynomial function (Figure 2). According to the temporal trend of publications and citations, the research period can be delineated into two phases: the first stage, preceding 2018, as a period of slow exploration, and the second stage, from 2018 to 2024, as a period of rapid development. Since 2018, global annual publications have exceeded 90, and citations have surpassed 2600 annually, showing a steady upward trend (Figure 2). Eighty disciplines have contributed to the research on ATTR-CM (Figure S1). The cardiovascular system was the dominant research field, followed by general internal medicine and molecular biology. Publications in these three disciplines accounted for more than half of all documents. ATTR-CM is also a target in experimental medicine, radiology, neurology, pharmacology, surgery, and genetics, which underscores the need for multidisciplinary collaborations to provide optimal disease management strategies.

### Research country analysis

Seventy-seven countries contributed to the research of ATTR-CM. For analytical purposes, Taiwan was included in the analysis of China, while Wales, Northern Ireland, and Scotland were grouped under the United Kingdom (UK). The top 10 countries with the most publications are listed in Table 1. The United States led with 705 publications (H-index 93), while seven among the top 10 countries were in Europe. Regarding annual publications, the combined output of the USA and Italy surpassed that of other countries worldwide (Figure 2). China, one of the two Asian countries and the only developing country on the list (Table 1), demonstrated characteristics of a late starter but showed continuous progress (Figure 2).

**Figure 3:**
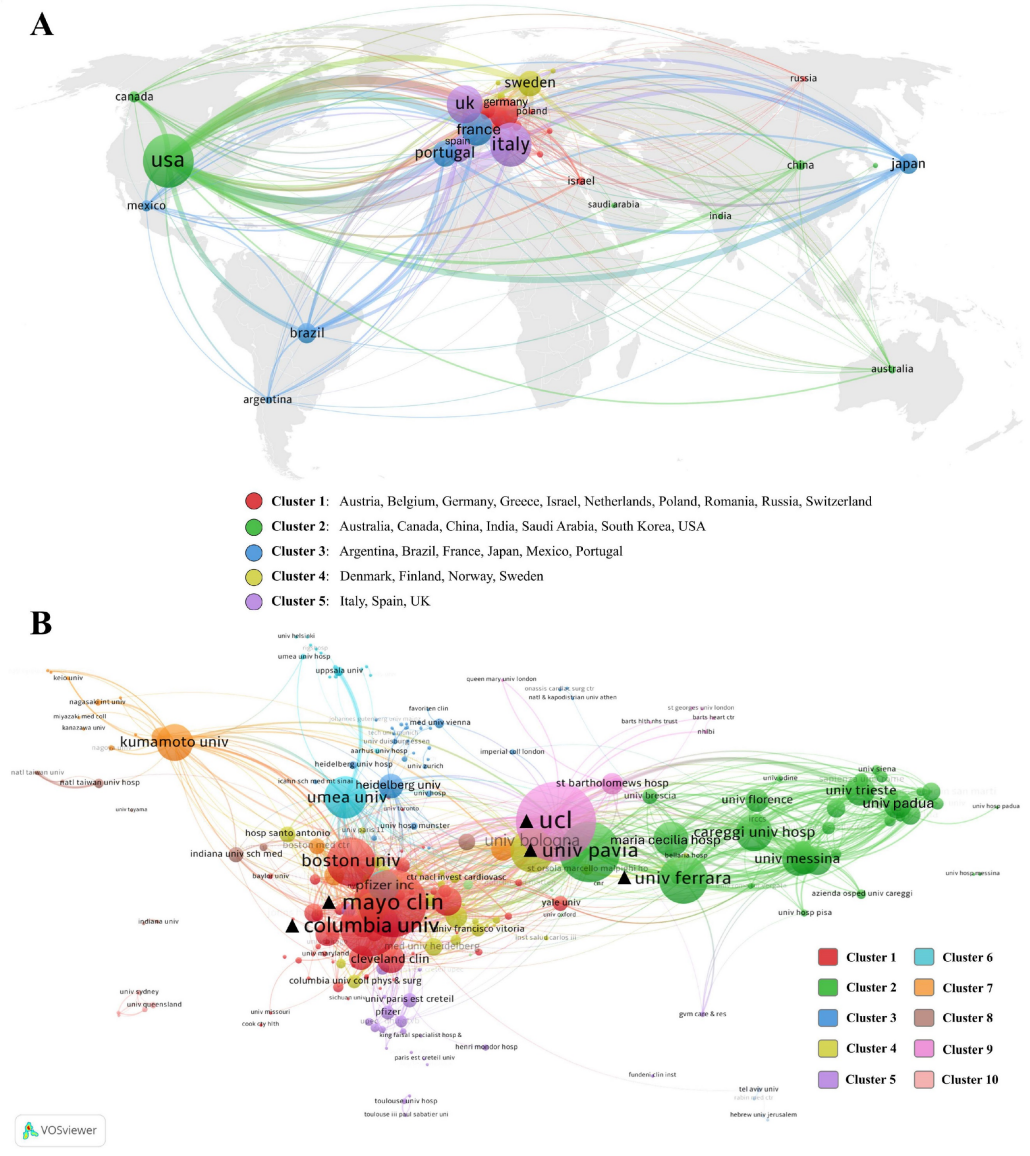
Collaboration networks of countries (A) and institutions (B).

In terms of collaboration levels, a co-authorship network and clustering of countries are visualized (Figure 3A). Based on collaboration frequency and publication volume, these countries are categorized into five distinct clusters. Cluster 1 predominantly comprised European nations, most of which were European Union member states. Cluster 2 spanned multiple continents. Despite the geographical distribution, the countries in Cluster 3 are rooted in shared historical, linguistic, and cultural backgrounds. Cluster 4 represented a typical Nordic collaboration cluster, while Cluster 5 consisted of developed countries in southwestern Europe. Beyond geographic proximity, the USA has the highest total link strength (TLS) and shows extensive collaboration across different country clusters and areas. Asian countries, including China, Japan, South Korea, India, and Saudi Arabia, lack comprehensive intra-continental and intercontinental synergy compared to the USA and European countries.

### Research institution analysis

A total of 2547 institutions were actively engaged in research on ATTR-CM. Among these, the top 10 institutions with the most publications are detailed in Table 1. Institutions from Europe and the United States dominated the list. Leading the list was the University of London, demonstrating remarkable productivity with 180 publications and an H-index of 60. It is noteworthy that the University of London operates as a university system, encompassing various colleges and research institutions, including University College London. In the realm of ATTR-CM, all articles published by University College London were affiliated with the University of London. This affiliation model was similarly observed in Assistance Publique - Hôpitaux Paris, Université Paris Est Créteil (UPEC), and Hopital Universitaire Henri Mondor, leading to shared publications among these institutions.

In terms of collaboration strength, a comprehensive network is illustrated in Figure 3B, with institutions divided into 10 clusters. University College London also exhibited the highest TLS, followed by Mayo Clinic and Columbia University. The University of Pavia and the University of Ferrara ranked 4_th_ and 5_th_, respectively. The clustering of institutions revealed a clear national pattern. Cluster 1 was primarily composed of medical centers and institutions from the United States, while Clusters 2 and 4 were dominated by Italian research institutions. Cluster 6 mainly consisted of institutions from Nordic countries, Cluster 7 was almost entirely composed of Japanese institutions.

### Authorial analysis

In the realm of ATTR-CM, a total of 9355 authors were engaged. According to Lotka's law, 185 authors who published 10 or more papers were considered core authors in this field due to their high productivity and leadership. These scholars accounted for approximately 1.98% of all authors but contributed 22.04% of the total publications. The top 10 prolific authors and their performance from several perspectives are listed in Table 2. The fractional individualized H-index (Hi-index) is a modification of the traditional H-index. Compared to the widely used H-index, it proportionally allocates contributions among co-authors, offering a more accurate reflection of the actual role of each researcher in collaborative work 15. Notably, seven of the top 10 most prolific authors hailed from Europe. Mathew S. Maurer was identified as the leading author, with 106 publications and an H-index of 47 (Hi-index of 21). Martha Grogan had the highest average citations per publication (ACP).

**Figure 4:**
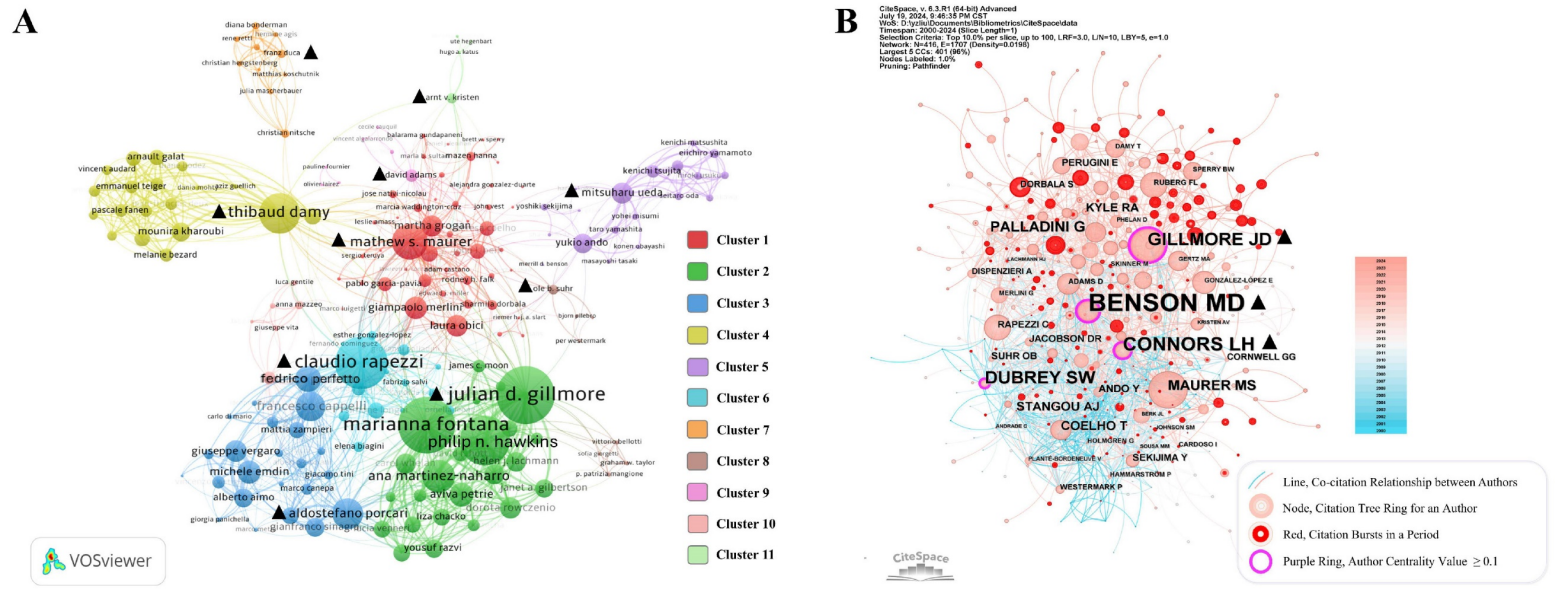
Networks of author collaborations (A) and co-cited authors (B).

The author collaboration network and clustering are visualized in Figure 4A. Authors are divided into 11 clusters, and the leading authors of each cluster are highlighted. Ole B. Suhr stood out as an early pioneer who laid the foundation for the field, while Marianna Fontana played a pivotal role in driving clinical translation during the intermediate phase. Aldostefano Porcari, representing the most recent cohort, emerged as a rising contributor in the field (Figure S2). The Co-citation relationship and network from references revealed that 9350 authors were co-cited. Merrill D. Benson had the highest centrality value at 0.17, followed by Julian D. Gillmore and Lawreen H. Connors (Figure 4B) in the co-citation network, suggesting their crucial role in connecting different research groups and leading emerging research topics.

### Journal analysis

A total of 441 journals published all the 1855 documents. According to Bradford's Law, the top 15 most prolific journals were considered core journals, as they published 644 papers, accounting for 34.7% of the total publications. The top 10 most productive journals are listed in Table 3, with their performance in other metrics also provided. Among these, *Amyloid: Journal of Protein Folding Disorders* had the most publications (173) and the highest H-index (40). *JACC: Cardiovascular Imaging* had the highest average citations per paper (80.33). Five of the top 10 journals were ranked in Q1 by Journal Impact Factor (JIF), while the remaining were in Q2. The Article Influence Score (AIS) evaluates a journal's quality and depth of impact in the academic field by measuring the influence and weight of its articles within the citation network, rather than relying solely on citation counts 16. *European Journal of Heart Failure* had the highest AIS and Impact Factor (IF) in the list. Additionally, four of these journals had a percentage of citable open-access (PCOA) articles exceeding 60%, indicating that open access publications may promote research progress in ATTR-CM by increasing the dissemination of research findings and accelerating the clinical translation of research results.

To explore the co-citation relationship and networks, 441 journals cited by 1855 papers were analyzed. The top 10 journals with the highest centrality values of all the references are also listed in Table 3. From this perspective, *Circulation* held the highest centrality value of 0.28, followed by *Neurology* (0.17),* Amyloid: Journal of Protein Folding Disorders* (0.14), and* Proceedings of the National Academy of Sciences of the United States of America* (0.14), reflecting their credibility and broad inclusivity across multiple disciplines. Nine of the top 10 journals were classified as Q1 by JCR rankings. In terms of disciplinary distribution, although these journals were primarily concentrated in the field of medicine, journals in neuroscience and hematology also held significant positions, indicating that studies on ATTR-related neuropathy and amyloid light chain amyloidosis have provided important insights and driven advancements in ATTR-CM research.

**Figure 5:**
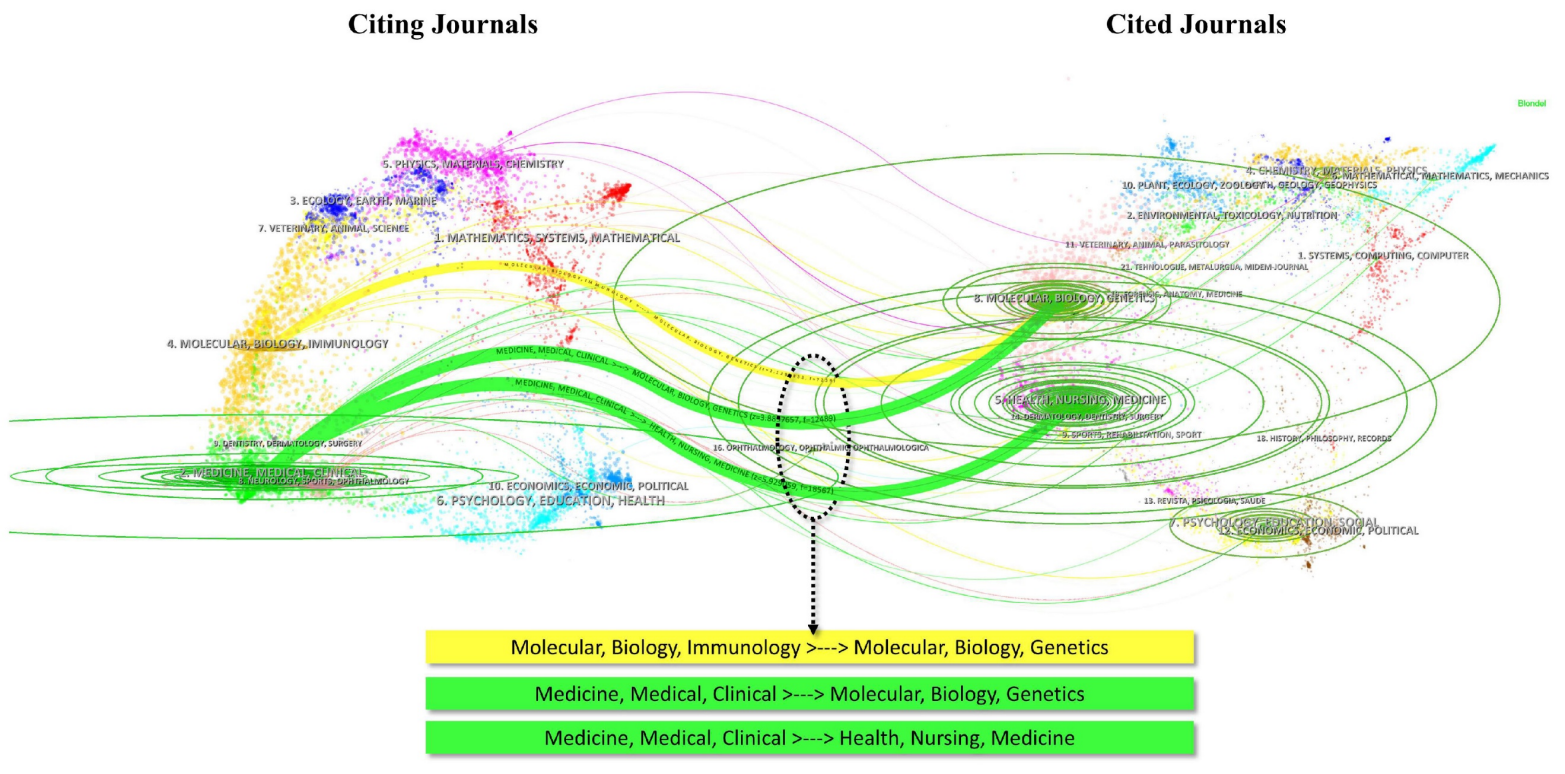
Dual-map overlay of journals. Curves represent citation pathways from citing journals to cited journals. Thickness of the curves indicates the frequency of citations.

The dual-map overlay of journals illustrates the distribution of literature across various disciplines and citation paths. In Figure 5, three main reference paths marked in yellow and green are evident. Studies in molecular biology and genetics were frequently cited by publications in molecular biology and immunology, whereas studies in medicine and clinical sciences not only cited literature from molecular biology and genetics but also from health, nursing, and medicine categories. These trajectories suggested a focus on molecular biological mechanisms and clinical medical translation.

### Publication and reference analysis

The top 10 most cited publications are presented in Table 4, with their primary contents detailed in Table S1. To comprehensively assess the impact of these research findings from multiple dimensions, refined indicators and qualitative measures were incorporated. The Altmetric Attention Score (AAS) captures the attention academic publications receive across non-traditional platforms, such as social media, news outlets, and blogs, complementing traditional bibliometric indicators by offering insights into the societal relevance and public engagement of research through a qualitative lens 17. The F1000 Article Factor (FFa), a weighted metric derived from expert recommendations on Faculty Opinions, assesses the quality and academic significance of research through qualitative peer review 18. The most cited literature was “Tafamidis Treatment for Patients with Transthyretin Amyloid Cardiomyopathy” 19. This article had the highest AAS (708), and FFa (14), highlighting its exceptional social and academic impact. Another noteworthy article, "Inotersen Treatment for Patients with Hereditary Transthyretin Amyloidosis" 20, had the highest FFa (14) and the second-highest AAS.

**Figure 6:**
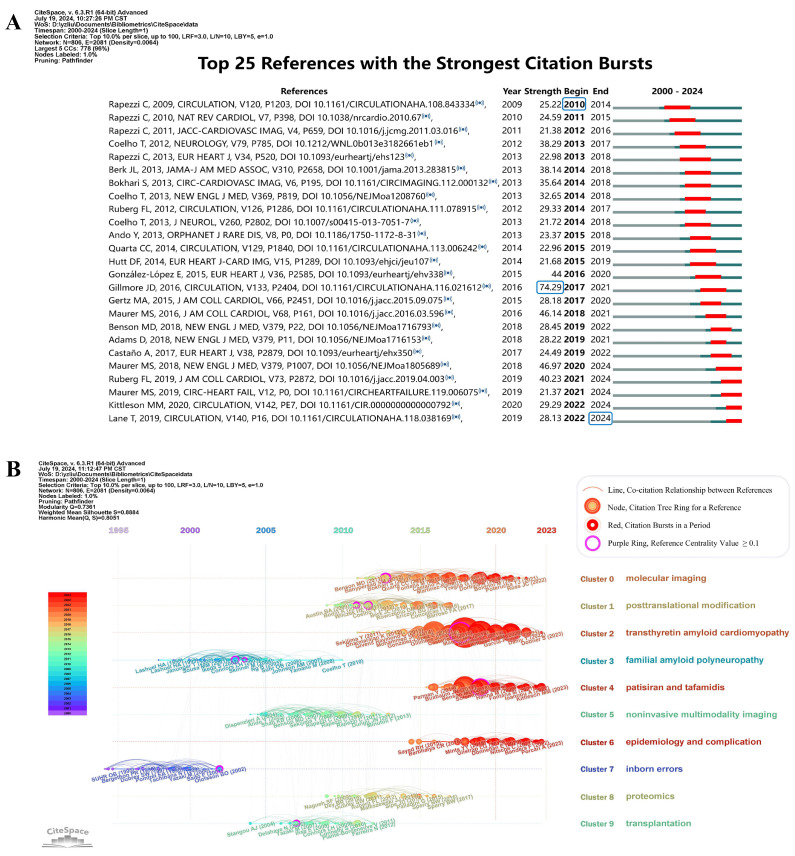
Visualization of co-cited references. (A) References with the strongest citation bursts. (B) Timeline and clustering of co-cited references.

To analyze the knowledge foundation and co-citation relationships underlying the 1,855 publications, 22,872 references were examined. Figure 6A presents the top 25 co-cited references with the strongest citation bursts, with their primary contents detailed in Table S2. The earliest reference in Figure 6A is *Systemic cardiac amyloidoses: disease profiles and clinical courses of the 3 main types* by Claudio Rapezzi *et al.*, published in 2009 21. The reference with the highest burst strength is *Nonbiopsy Diagnosis of Cardiac Transthyretin Amyloidosis* by Julian D. Gillmore *et al.*, published in *Circulation* in 2006, with a citation burst strength of 74.29 22. The latest reference in Figure 6A is *Natural History, Quality of Life, and Outcome in Cardiac Transthyretin Amyloidosis* by Thirusha Lane *et al.*, published in 2019 23. References with high centrality, shown in Figure S3, have influenced and been cited by studies across various fields, establishing their central positions spanning multiple disciplines. The reference with the highest centrality value (0.34) is *Progressive wild-type transthyretin deposition after liver transplantation preferentially occurs in myocardium in FAP patients*, published in 2007 in *American Journal of Transplantation* by Masahide Yazaki *et al.* 24, followed by *Variation in amount of wild-type transthyretin in different fibril and tissue types in ATTR amyloidosis*, published in 2010 by Elisabet Ihse *et al.* in the *Journal of molecular medicine* 25.

To further trace the development trajectories in different facets of ATTR-CM, Figure 6B presents a timeline visualization of the 10 largest clusters. The cited references cluster network exhibits a logical and well-defined community structure, with Modularity Q = 0.74 (> 0.3) and Silhouette value = 0.89 (> 0.7). Cluster 7 (inborn errors) and Cluster 3 (familial amyloid polyneuropathy) emerged earliest on the timeline map, suggesting an initial focus on familial ATTR amyloidosis on neuropathy 21. Conversely, Clusters 2 (transthyretin amyloid cardiomyopathy), Clusters 4 (patisiran and tafamidis), Clusters 6 (epidemiology and complication), and Cluster 0 (molecular imaging) represented currently prominent topics of interest.

### Keyword analysis

**Figure 7:**
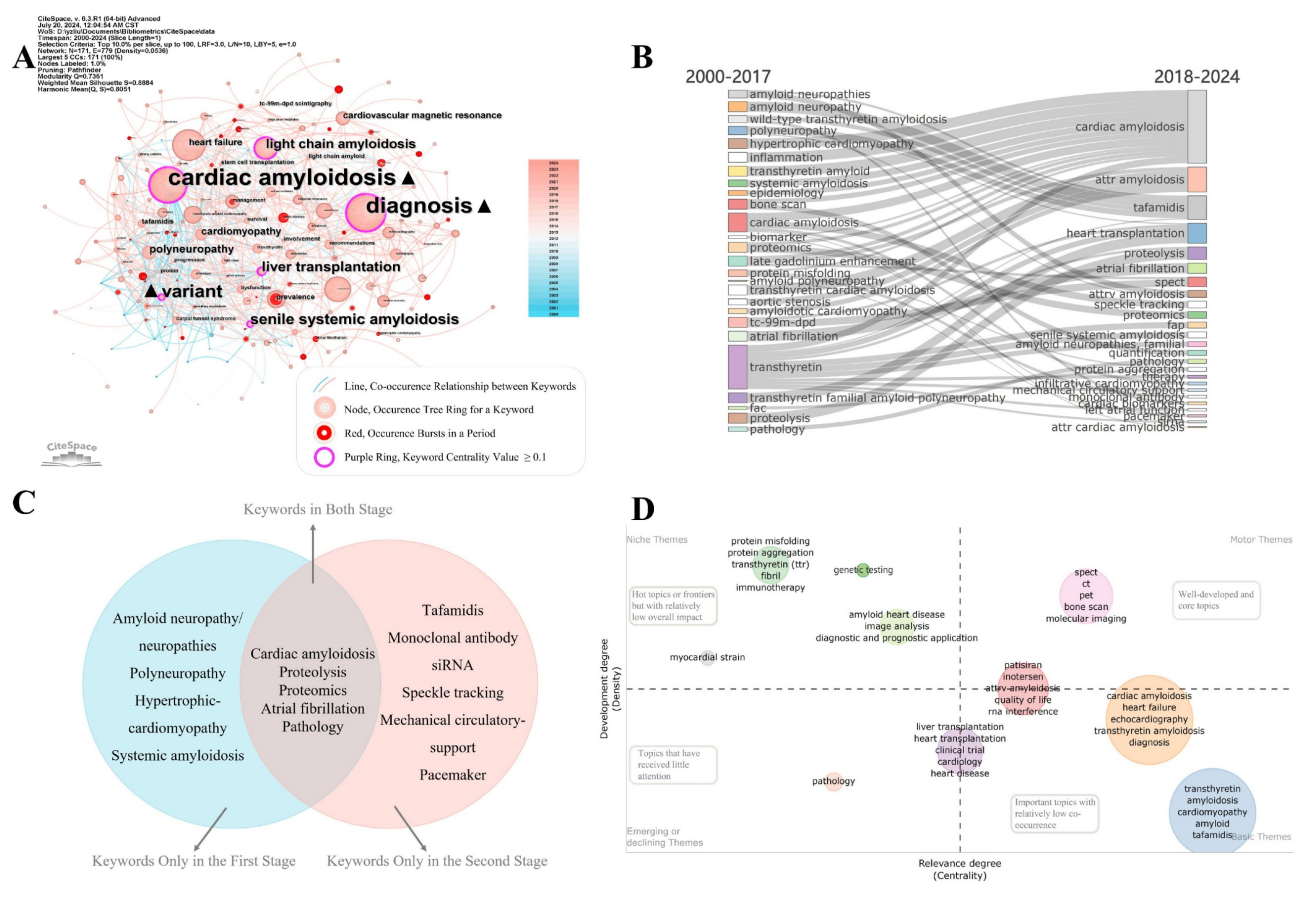
Keyword analysis. (A) Co-occurrence network of keywords. (B) Keyword evolution across two periods. (C) Venn diagram showing three categories of keywords across two periods. (D) Classification of research themes.

Developmental trends and research focal points in ATTR-CM were delineated through the keyword analysis (Figure 7). Data standardization was performed to mitigate discrepancies arising from subtle terminological differences. For instance, “ATTR-CM”, “ATTR-CA”, “transthyretin cardiac amyloidosis”, and “ATTR cardiomyopathy” were merged into “transthyretin amyloid cardiomyopathy”. The keyword co-occurrence network is shown in Figure 7A, and the top 25 keywords with the highest co-occurrence frequency are listed in Table S3. "Cardiac amyloidosis”, followed by "diagnosis" emerged as the most frequently occurring keywords and those with the highest centralities. Other keywords, including "variant", "liver transplantation", "light chain amyloidosis", and "polyneuropathy", had centrality values of 0.1 or greater. These keywords underscore the key focal points in the research field and serve as bridging elements within the interdisciplinary research network of ATTR-CM.

Research themes during the slow exploration and rapid development periods were analyzed, and key topics are illustrated in the Sankey diagram (Figure 7B), highlighting both continuity and change between the two stages (Figure 7C). "Cardiac amyloidosis”, "proteolysis”, "proteomics”, "atrial fibrillation”, and "pathology" appear in both stages, indicating continuous attention to basic sciences, complication management, and pathological mechanisms. Changes include the attrition of certain keywords and the emergence of novel ones in the second stage. Terms including "amyloid neuropathy/neuropathies”, "polyneuropathy”, "hypertrophic cardiomyopathy”, and "systemic amyloidosis" are absorbed into other keywords in the second stage, reflecting a refinement on ATTR-CM. Novel keywords in the second stage include "tafamidis", "monoclonal antibody", "siRNA", "speckle tracking", "mechanical circulatory support", and "pacemaker", reflecting a significant shift towards novel therapeutics, advanced imaging modalities, and life support technologies (Figure 7C).

Research topics were further classified based on their centrality and density, which were presented as a coordinate plot. The thematic quadrant mapping (Figure 7D) categorizes research topics into four quadrants to show the relevance and development degree of the topics. Niche themes, which are hot topics and frontiers in specific areas but with relatively low overall impact, are mainly associated with molecular biology and diagnostic/prognostic algorithms. Motor themes, which are well-developed and core topics, primarily encompass imaging methods but also include RNA interference medications such as patisiran and inotersen. Basic themes are important topics with relatively low co-occurrence, encompassing heart failure, heart or liver transplantation, and tafamidis, which require significant funding, clinical ethics approval, and long-term clinical trials. Emerging theme is mainly about pathology, which is not fully understood.

## Discussion

### Knowledge landscape of studies in ATTR-CM

ATTR-CM is an infiltrative cardiomyopathy resulting from the accumulation of misfolded TTR. Cardiomyopathy is the main cause of death in patients with ATTR amyloidosis due to arrhythmia, valve stenosis, and progressive heart failure 26. However, misdiagnosis or delayed diagnosis results in relatively poor quality of life and prognosis 23. In this study, the knowledge landscape and development trajectory of ATTR-CM were elucidated to foster deeper research in this field.

Multiple disciplines have contributed to the expansion of knowledge on ATTR-CM, and the publication trend indicates a continuing upward trajectory (Figure S1 and Figure 2). This trend can be attributed to the increasing prevalence and clinical awareness of ATTR-CM, driven by an aging population and heightened awareness of its impact on life expectancy in the elderly. Advances in diagnosis and treatment, achieved through multidisciplinary research efforts, have established a significant positive feedback mechanism, as evidenced by the growing volume of publications in this field. The year 2018 marked a pivotal transition from the period of slow exploration (the first stage) to rapid development (the second stage), driven by the success of several large-scale clinical trials of TTR-targeted therapies, including tafamidis, inotersen, and patisiran 19, 20, 27. The advancement of gene testing and editing technologies serve as another major catalyst, facilitating significant progress in early diagnosis and promoting the development of gene therapies for this disease 28. The application of these technologies has enabled the identification of a broader patient population and supported the investigation of associations between genetic backgrounds and disease phenotypes.

The analysis at country, institution, and author levels identified that Europe and the USA dominate the field of ATTR-CM research. Asian and African countries still have room for improving international synergy compared to the western countries (Figure 2 and Figure 3A). International collaboration is influenced by geographic location, language, culture and historical ties, driving trends of globalization and regional intensification (Figure 3A). Globalization fosters cross-border partnerships on global challenges, supported by technological advances and large-scale funding. Simultaneously, regional intensification strengthens collaborations among neighboring or culturally aligned countries, leveraging proximity and commonalities in communication, clinical and genetic backgrounds, and healthcare policies. These dynamics enhance knowledge exchange but also illustrate disparities in resources, emphasizing the need for more inclusive collaborations. Institutional collaboration clustering demonstrates a clear national dependency (Figure 3B), likely driven by domestic or regional grants and policy support. Factors such as the ease of sharing clinical and research resources, alignment of healthcare systems, and focused national efforts on ATTR-CM further reinforce this trend. In the co-authorship network, the representatives of each cluster are typically the most prolific authors (Figure 4A and Table 3), suggesting that extensive collaboration contributes to high productivity, which in turn reinforces their authoritative standing in the field.

The analysis of journals, documents, and their references unveiled the knowledge foundation and landmark advancements in the realm of ATTR-CM. The top 10 journals in publication numbers and as sources of cited references are of high quality, with open access promoting the dissemination and translation of research outcomes (Table 3). By clustering the references, ten main themes in ATTR-CM research are summarized (Figure 6B). The evolution of research themes in ATTR-CM reveals that, while significant attention has been given to pathophysiological mechanisms and complication management, transformative breakthroughs have been scarce. This underscores the necessity of aligning research designs more closely with pressing clinical challenges and leveraging emerging technologies. As the focus narrows from general amyloidosis to specific subfields within ATTR-CM (Figure 7B), interdisciplinary collaboration becomes crucial, fostering both knowledge expansion and the emergence of innovative research directions.

### Research hotspots and future directions in ATTR-CM

#### Early diagnosis and prognosis algorithm

In this study, three of the ten most-cited articles address diagnostic techniques for ATTR-CM, and one of the articles is also the reference with the highest citation bursts (Table 4 and Figure 6A). Results from reference and keywords analysis also underscore its importance (Figure 6B and 7D). Nuclear scintigraphy using bone avid radiotracers has been proven to have high specificity and positive predictive value, making it the only imaging modality capable of accurately diagnosing ATTR-CM without a cardiac biopsy 22. However, this technique also has its limitations in application and provides no prognostic information 29. No biomarker is specific or reliable enough to predict ATTR-CM, though many multiparametric biomarker-based prognostic scores have been proposed. Julian D. Gillmore *et al.* provided a staging system for ATTR-CM patients, showing a negative correlation between stage and prognosis in 2018 30. Cheng *et al.* proposed a new stage system using Mayo or NAC scores, the dose of furosemide or equivalent, and NYHA class 31, 32. These systems need validation in clinical trials to assess their effectiveness. Circulating TTR and retinol-binding protein 4 (RBP4) and plasma neurofilament light chains (NfL) were considered to be promising substitutes for cardiac troponin I (cTnI) or NT-proBNP 33, 34, but their efficacies remain unconfirmed in population studies and they have yet to be implemented in clinical practice.

It is reasonable to expect a simple and feasible non-invasive screening method or prognostic prediction model by integrating manifestations, imaging information, and biomarkers 5, 35. Such evaluations are crucial for optimizing clinical implementation, informing evidence-based guidelines, and enhancing diagnostic capabilities across healthcare settings. Further research is needed to develop robust risk prediction frameworks, establish effective long-term follow-up strategies, while critically evaluating the feasibility, cost-effectiveness, and clinical utility of these diagnostic algorithms and prognostic models through clinical trials and real-world evidence.

#### TTR-specific medication development

From both academic and societal impact perspectives, medication development is one of the most prominent themes in the field of ATTR-CM, since four of the top 10 most-cited articles are related to new medication development and have high AAS and FFa scores (Table 4 and Table S1). In addition, the reference analysis and evolution of research themes underscore the innovative and transformative potential of TTR-specific medications (Figures 6B and 7C), which may alter the natural course of ATTR-CM and improve patient survival.

By far, TTR-specific medications can be divided into four categories: TTR-stabilizer exemplified by tafamidis, TTR-silencer including ASO and small interfering RNA (siRNA), TTR-modifier based on CRISPR-Cas9 technology, and TTR-remover by monoclonal antibodies. Tafamidis and Acoramidis stabilize the TTR tetramer. Studies shown that Tafamidis and Acoramidis reduced all-cause mortality, cardiovascular-related hospitalizations, and reduced the decline in functional capacity and quality of life 19, 36, 37. Higher levels of NT-proBNP at the time of diagnosis were associated with a poorer tafamidis treatment response 38, highlighting the importance of timely initiation. TTR-silencer, Inotersen, a 2'-O-methoxyethyl-modified phosphorothioate ASO, silences TTR protein by promoting mRNA degradation. It was shown to improve the mean left ventricular mass measured by CMR imaging and the performance in the six-minute walk test in polyneuropathy patients with ATTRv amyloidosis 20. However, side effects such as thrombocytopenia, hemorrhage, and renal impairment, cannot be ignored. Eplontersen (AKCEA-TTR-LRx) is an ASO with targeted delivery systems, designed to enhance cellular uptake and reduce systemic toxicity. Phase 3 clinical trials of it for ATTR-CM are currently underway (EPIC-ATTR study, NCT06194825, and CARDIO-TTRansform study, NCT04136171). Patisiran, an siRNA therapeutic agent, inhibits the production of hepatic TTR by forming an RNA-induced silencing complex leading to mRNA degradation. The APOLLO-B study revealed that patisiran slowed the decline in terms of functional and quality of life metrics in ATTR-CM patients, though no significant benefits were observed in mortality or hospitalization 39. The recently published HELIOS-B study demonstrated that vutrisiran, another siRNA agent, reduces the risk of all-cause mortality and recurrent cardiovascular events in patients with ATTR-CM 40. A TTR-modifier agent aims to knock down TTR genes using CRISPR-Cas9 and is underway (NCT04601051) 41. Despite promising results from *ex vivo* experiments 42, there has been limited evidence supporting the use of TTR-removers. A phase 2 study evaluating anti-serum amyloid P treatment for reducing cardiac amyloid load in ATTR-CM was terminated due to an unfavorable benefit-risk profile (NCT03044353). Another monoclonal antibody study was also terminated due to the impact of the COVID-19 pandemic (NCT03336580).

Currently, new medications are typically evaluated in the ATTR-CM population only after demonstrating efficacy in treating neuropathy in ATTR amyloidosis, delaying clinical translation. Clinical studies should be initiated earlier in ATTR-CM patients, and parallel research is recommended on both polyneuropathy and cardiomyopathy. Future studies should investegate the long-term efficacy, safety, and effectiveness of combination therapies, while also exploring outcome variations across subgroups. This approach will support the development of more precise and personalized treatment strategies. A further goal is to explore the potential to reverse the pathological progression of ATTR-CM, paving the way for long-term, sustainable disease control.

#### Management of comorbidities and complications

Complications such as heart failure, atrial fibrillation, and aortic stenosis are prominent in the reference and keyword analysis (Figures 6B, 7A, and 7C). This underlines the urgent and cutting-edge status of comorbidities and complication research in the ATTR-CM field.

Few agents have shown improvements in the prognosis of heart failure aside from TTR-specific therapies, due to the infiltrative nature of ATTR-CM, which reduces stroke volume, decreases compliance, and impairs cardiac output 43. The standard four pillars of heart failure management may have limitations when applied to ATTR-CM. Beta-blockers may be harmful due to their negative chronotropic and inotropic effects 44, although observational studies suggested low doses may reduce the risk of mortality in ATTR-CM patients with heart failure 45, 46. Patients with ATTR-CM exhibit neurohormonal activation, but the use of renin-angiotensin-aldosterone system inhibitors (RAASi) is associated with an increased risk of hypotension 47. Mineralocorticoid receptor antagonists (MRA) are rarely discontinued and are linked to a reduced risk of mortality 45. Cohort studies indicated good tolerance of SGLT2 inhibitors in ATTR-CM patients 48, 49. A completed phase 4 trial assessing the safety and tolerability of empagliflozin in ATTR-CM-related heart failure (NCT05233163) has yet to report results. As a result, clinical practice is primarily based on expert consensus. Arrhythmia requires further analysis in the context of ATTR-CM 50. Atrial fibrillation is more prevalent in patients with ATTR-CM compared to those with light chain cardiac amyloidosis and the general population 51. Given the lack of association between the CHA_2_DS_2_-VASc risk score and the presence of intracardiac thrombosis in ATTR-CM patients with atrial fibrillation 52, it is recommended to initiate anticoagulation therapy upon the diagnosis of atrial fibrillation in these patients. Whether vitamin K antagonists or direct oral anticoagulants are more effective in preventing thrombosis lacks sufficient supporting data. Treatment for atrial fibrillation in ATTR-CM is challenging. It is needed to weigh the benefits between rhythm control and ventricular rate control 53, 54. The indication for implantable cardioverter defibrillator (ICD) for primary prevention of ventricular tachycardia remains controversial 55. Valve stenosis is common in ATTR-CM, with transcatheter aortic valve replacement (TAVR) suggested to be superior to surgical aortic valve replacement for aortic stenosis 56. However, there is no preference for mitral and tricuspid valve diseases.

In the management of heart failure, future research should focus on the indications for, dose optimization, and combination therapy of standard heart failure treatments in ATTR-CM patients. Regarding arrhythmia management, more clinical trials are needed to guide clinical decision-making. In valve disease management, future research should focus on improving surgical procedures and postoperative care to maximize patient outcomes. Optimizing comorbidity management is essential for improving quality of life across all stages of ATTR-CM. However, conducting clinical trials in this context often faces challenges such as difficulty in controlling participant risk, baseline imbalances among enrolled subjects, and ethical constraints. Simulated clinical trials may provide preliminary evidence and valuable references to support real-world studies and traditional clinical trials.

#### Epidemiology and genotype-phenotype correlation

Penetrance, prognosis, and regional distribution in patients with ATTRv amyloidosis vary with different single amino acid mutations in *TTR* gene. Therefore, the epidemiology and genotype-phenotype relationship remain unclear 2. Epidemiology is a key research cluster (Figure 6B), while "variant" and "genetic testing" are prominently highlighted in the keyword analysis (Figures 7A and 7D, and Table S3). These findings highlight the importance of further investigation in these areas.

With the advancement of next-generation sequencing, genetic testing has facilitated the diagnosis of ATTR amyloidosis and provided feasibility for clarifying the epidemic characteristics and genotype-phenotype correlations. Currently, the most common mutation type worldwide is Val30Met 57. This genotype is prevalent in Sweden, Portugal, and Japan. Though initially recognized as the prototype of familial polyneuropathy, it also manifests as cardiomyopathy 2. The most common genotype in the USA is Val122Ile (pV142I), predominantly observed in patients of Western African origin, with a prevalence of nearly 3.4%. It shows a male predominance and leads to delayed restrictive cardiomyopathy without neuropathy 58. The median survival for untreated Val122Ile patients after diagnosis is estimated at 2.5 years, the shortest among those with ATTRwt amyloidosis and other types of ATTRv amyloidosis. This outcome has been shown to be associated with race and socioeconomic status 59.

Future research could explore how genetic mutations interact with race, gender, and socioeconomic factors, and evaluate the correlation of these factors with clinical outcomes. With advancements in non-invasive diagnosis and gene testing technology, it is now possible to more precisely estimate the incidence or prevalence, penetrance, and genotype-phenotype relationships worldwide through comprehensive cooperation, especially in regions like Asia and Africa. This will provide essential evidence for identifying high-risk patients, improving prognosis, and advancing targeted treatment strategies.

#### Molecular biology and mechanisms

Molecular biology and biochemical mechanisms provide basic theory of pathogenesis and serve as a blueprint role for guiding treatment revolution in the future. In this study, proteomics is identified as a major research cluster (Figure 6B), while the keywords "protein aggregation" and "protein misfolding" are identified as niche research themes (Figure 7D), indicating their potential for further exploration.

Although the primary through quaternary protein structure of fibrillary precursor was identified, the precise mechanisms by which structural changes lead to amyloid fiber formation remain unclear 60. The question of why normal TTR can cause tissue deposition of amyloid fibrils is not well understood. Researchers believe that proteolysis might play a crucial role in pathogenesis, and intriguingly, some kinetic stabilizers are effective in preventing proteolysis and subsequent fibrillization of TTR 61. Although experiments targeting specific proteases are significant, they have yet to yield conclusive evidence 62, 63. Another question is that the stability of TTR tetramer does not solely determine amyloid deposits. For instance, the Val122Ile tetramer is thermodynamically unstable, but amyloidosis develops later in life 64. An experiment in cellular mechanisms described a novel endoplasmic reticulum quality control bypass mechanism. It revealed that the secretion of monomeric forms of late-onset *TTR* mutations contributes to amyloid formation, partly explaining the variable onset of ATTR-CM 65. In ATTRv amyloidosis patients, the varying proportions of normal and mutated TTR deposition in different tissues warrant further investigation to clarify these differences.

Future research may uncover new breakthroughs by integrating molecular mechanisms with other fields, expanding the influence and applicability of ATTR amyloidosis studies. Proteomics, supported by artificial intelligence-assisted machine learning techniques, could shed light on the underlying pathogenic mechanisms of ATTR amyloidosis 66. Interdisciplinary collaboration between molecular biology and computational biology holds the potential to uncover previously unknown molecular mechanisms, identify new therapeutic targets, and drive transformative advancements in the treatment of ATTR amyloidosis.

### Advantages and limitations

This study is the first bibliometric analysis of ATTR-CM research over the past 24 years. By incorporating qualitative assessments to mitigate the limitations of citation metrics, this study provides a more comprehensive and objective evaluation of the knowledge landscape and research hotspots in the field. Building on these findings, targeted perspectives and approaches for future exploration are proposed, offering insights to advance scientific research and enhance clinical practice.

This study has several limitations. First, to ensure data consistency and comparability, we relied exclusively on the WoSCC database for its representativeness, high quality, and comprehensive multidisciplinary coverage compared to other databases 67. Nonetheless, some relevant literature from other databases might not have been included. Second, only English-language literature was included, which may introduce selection bias by excluding important research from other languages, particularly European studies. However, research from non-English-speaking European countries is well-represented in the English-language publications included in this analysis, suggesting that language bias has a limited impact on the findings. Third, papers published before 2000 were excluded to ensure data relevance and accuracy, which may limit the historical context. Nevertheless, this study suggests that the impact of literature prior to 2010 is minimal, and reference analysis helped mitigate this limitation. Lastly, the use of automated algorithms in bibliometric tools may introduce methodological biases, which should be considered when interpreting the findings.

## Conclusions

This bibliometric analysis is the first comprehensive exploration of the knowledge landscape in ATTR-CM research over the past 24 years. It constructs a detailed knowledge map that identifies influential academic entities and communities, pivotal literature, and impactful journals based on academic impact, societal influence, and disciplinary centrality. Additionally, it reveals major research themes, uncovers five current research hotspots, and outlines future directions and approaches. By enhancing awareness and providing a deeper understanding of ATTR-CM, this analysis offers valuable insights that help drive advancements in scientific research and clinical practice, ultimately contributing to improved patient outcomes.

## Supplementary Material

Supplementary figures and tables.

## Figures and Tables

**Table 1:** Top 10 most productive countries and institutions

Rank	Country	NP	ACP	H-Index	Institution	NP	ACP	H-Index
1	USA	705	47.6	93	University of London	180	84.39	60
2	Italy	367	51.34	63	University College London	171	87.59	59
3	UK	218	75.64	61	Assistance Publique - Hôpitaux de Paris (APHP)	138	88.56	50
4	Japan	207	31.16	41	Columbia University	120	98.03	47
5	France	180	72.51	53	Mayo Clinic	113	97.59	45
6	Germany	139	67.86	46	Harvard University	108	68.28	38
7	Sweden	116	63.52	45	Université Paris Est Créteil -Val-de-Marne (UPEC)	96	101.13	38
8	Spain	113	43.89	27	Hopital Universitaire Henri Mondor (APHP)	95	102.14	38
9	China	90	5.98	14	Kumamoto University	89	36.67	29
10	Portugal	84	77.69	38	Boston University	86	99.3	38
					Umea University	86	61.74	40

NP, Number of publications; ACP, Average citations per publication.

**Table 2:** Top 10 most prolific authors and co-cited authors

Rank	Prolific Author	NP	ACP	H-Index	H_i_-Index	Co-cited Author	Centrality
1	Mathew S. Maurer	106	99.6	47	21	Merrill D. Benson	0.17
2	Philip N. Hawkins	79	100.62	41	17	Lawreen H. Connors	0.13
3	Marianna Fontana	78	88.71	35	16	Julian D. Gillmore	0.12
4	Thibaud Damy	77	90.57	30	12	Simon W. Dubrey	0.12
5	Julian D. Gillmore	67	107.75	34	17	Giovanni Palladini	0.1
6	Claudio Rapezzi	66	98.09	32	11	Mathew S. Maurer	0.09
7	Mitsuharu Ueda	57	22.42	20	7	Teresa Coelho	0.08
8	Federico Perfetto	49	22.33	19	5	Robert A. Kyle	0.08
9	Martha Grogan	49	143.92	27	14	Arie J. Stangou	0.08
10	Ole B. Suhr	49	63.49	31	14	Claudio Rapezzi	0.07

NP, Number of publications; ACP, Average citations per publication; Hi-Index: fractional individualized H-index.

**Table 3:** Top 10 most productive journals and co-cited journals

Rank	Publish Journal	NP	ACP	JCR rankings	IF (2023)	AIS	PCOA	Co-cited Journal	Centrality	JCR rankings	IF (2023)	AIS
1	*Amyloid: Journal of Protein Folding Disorders*	173	27.23	Q1	5.2	2.5	31.25%	*Circulation*	0.28	Q1	35.5	13.8
2	*Esc Heart Failure*	71	11.63	Q2	3.2	1.2	67.59%	*Neurology*	0.17	Q1	7.7	3.2
3	*Journal of Nuclear Cardiology*	64	20.69	Q2	3.0	0.8	21.52%	*Amyloid: Journal of Protein Folding Disorders*	0.14	Q1	5.2	2.5
4	*European Journal of Heart Failure*	38	61.32	Q1	16.9	6.0	49.38%	*Proceedings of the National Academy of Sciences of the United States of America*	0.14	Q1	9.4	4.4
5	*Journal of Clinical Medicine*	36	3.78	Q1	3.0	0.8	99.73%	*Blood*	0.13	Q1	21.0	7.4
6	*Frontiers in Cardiovascular Medicine*	35	4.71	Q2	2.8	0.8	99.58%	*New England Journal of Medicine*	0.12	Q1	96.2	41.5
7	*American Journal of Cardiology*	32	12.56	Q2	2.3	0.9	14.56%	*Journal of the American College of Cardiology*	0.11	Q1	21.7	10.2
8	*JACC: Cardiovascular Imaging*	30	80.33	Q1	12.8	5.2	71.73%	*Haematologica*	0.10	Q1	8.2	3.2
9	*International Journal of Cardiology*	29	13.55	Q2	3.2	1.0	20.14%	*American Journal of Cardiology*	0.09	Q2	2.3	0.9
10	*Heart Failure Reviews*	26	20.92	Q1	4.5	1.2	26.84%	*American Journal of Medicine*	0.08	Q1	5.1	1.8
								*Brain*	0.08	Q1	10.6	4.7

NP, Number of publications; ACP, Average citations per publication; JCR, Journal citation reports; IF, Impact factor; AIS, Article influence score; PCOA, Percentage of citable open access.

**Table 4:** Top 10 most cited publications

Rank	Publication Title	DOI	NC	ACY	AAS	FFa
1	*Tafamidis Treatment for Patients with Transthyretin Amyloid Cardiomyopathy*	10.1056/NEJMoa1805689	1420	202.86	708	14
2	*Nonbiopsy Diagnosis of Cardiac Transthyretin Amyloidosis*	10.1161/CIRCULATIONAHA.116.021612	1208	134.22	44	9
3	*Inotersen Treatment for Patients with Hereditary Transthyretin Amyloidosis*	10.1056/NEJMoa1716793	912	130.29	644	14
4	*Wild-type transthyretin amyloidosis as a cause of heart failure with preserved ejection fraction*	10.1093/eurheartj/ehv338	708	70.8	58	-
5	*Systemic amyloidosis*	10.1016/S0140-6736(15)01274-X	589	65.44	26	-
6	*Noninvasive etiologic diagnosis of cardiac amyloidosis using ^99m^Tc-3,3-diphosphono-1,2-propanodicarboxylic acid scintigraphy*	10.1016/j.jacc.2005.05.073	587	29.35	14	-
7	*Transthyretin Amyloid Cardiomyopathy: State-of-the-Art Review*	10.1016/j.jacc.2019.04.003	548	91.33	123	6
8	*Systemic Cardiac Amyloidoses Disease Profiles and Clinical Courses of the 3 Main Types*	10.1161/CIRCULATIONAHA.108.843334	534	33.38	16	-
9	*Tafamidis, a potent and selective transthyretin kinetic stabilizer that inhibits the amyloid cascade*	10.1073/pnas.1121005109	530	40.77	116	-
10	*Diagnosis and treatment of cardiac amyloidosis. A position statement of the European Society of Cardiology Working Group on Myocardial and Pericardial Diseases*	10.1002/ejhf.2140	483	120.75	226	-

NC, Number of citations; ACY, Average citations per year; AAS: Almetric attention score; FFa: F1000 article factor.

## Abbreviations

ACP: Average Citation per Publication
AIS: Article Influence Score
AAS: Altmetric Attention Score
ASO: Antisense Oligonucleotide
ATTR amyloidosis: Transthyretin Amyloidosis
ATTR-CA: Transthyretin Cardiac Amyloidosis
ATTR-CM: Transthyretin Amyloid Cardiomyopathy
ATTRv: Variant Transthyretin Amyloid
ATTRwt: Wild-type Transthyretin Amyloid
cTnI: Cardiac Troponin I
eGFR: Estimated Glomerular Filtration Rate
FFa: F1000 Article Factor
HFpEF: Heart Failure with Preserved Ejection Fraction
Hi-index: individualized H-index
IF: Impact Factor
JCR: Journal Citation Reports
LVEF: Left Ventricular Ejection Fraction
MRA: Mineralocorticoid Receptor Antagonists
NC: Number of Citations
NfL: Neurofilament Light Chains
NP: Number of Publications
NT-proBNP: N-terminal pro B-type Natriuretic Peptide
PCOA: Percentage of Citable Open Access
RAASi: Renin-Angiotensin-Aldosterone System Inhibitors
RBP4: Retinol-binding Protein 4
SGLT-2i: Sodium-Glucose Cotransporter 2 Inhibitors
siRNA: Small Interfering RNA
TAVR: Transcatheter Aortic Valve Replacement
TS: Topic Search
TTR: Transthyretin
UPEC: Université Paris Est Créteil
USA: United States of America
WoSCC: Web of Science Core Collection
