# Supplementary material for: Knowledge Landscape and Hotspots of Research in Transthyretin Amyloid Cardiomyopathy: A Bibliometric Analysis
Source: Int J Med Sci. 2025 Feb 28;22(7):1585–601. doi: 10.7150/ijms.101888 (PMC11905275; doi:10.7150/ijms.101888)
Supplement: Supplementary file 1 — Supplementary figures and tables. [file ijmsv22p1585s1.pdf]

Supplementary Figures

Figure S1

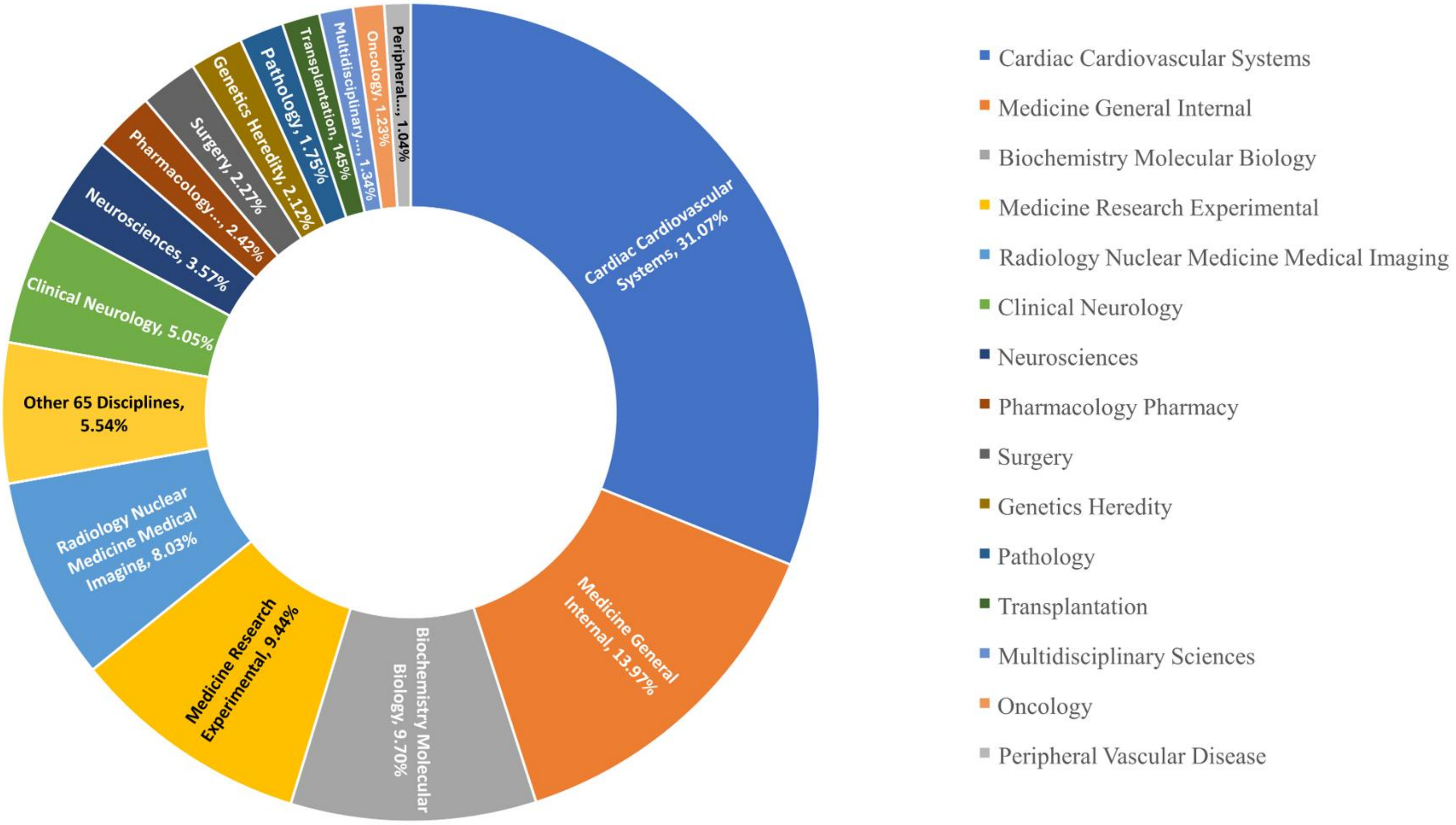

Figure S1. Disciplines in ATTR-CM research.

Figure S2

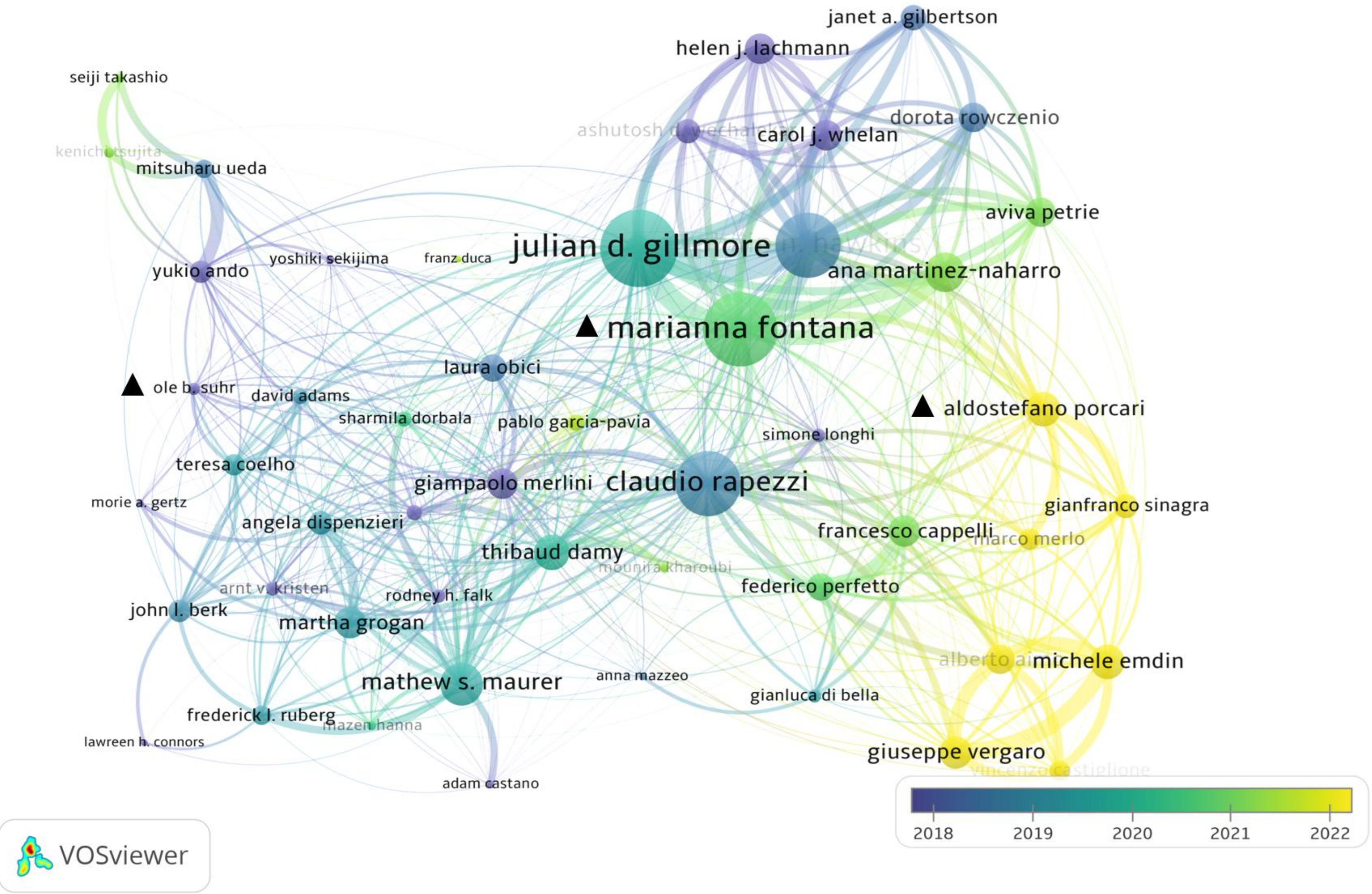

Figure S2. Collaboration network among the top 50 prolific authors. Node and label size represent the total link strength of each author. Link thickness indicates the frequency of collaboration. Colors correspond to the weighted average publication year of each author.

Figure S3

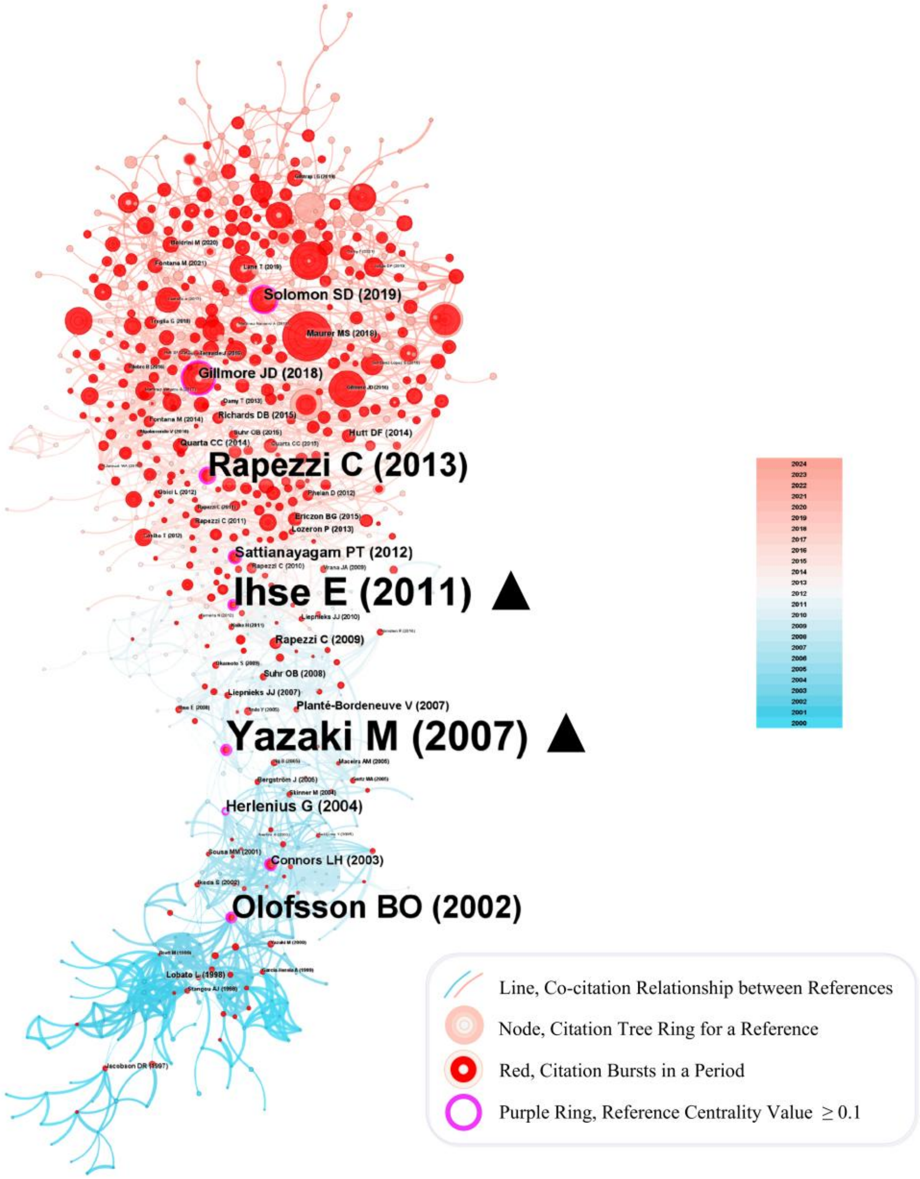

Figure S3. Co-citation network of references. Node size represents co-citation frequency. Label size reflects proportional centrality values. Tree-ring node and line colors indicate the timing of co-citation relationships.

Supplementary Tables

Table S1

Primary content of the top 10 cited publications

Rank	Publication Title	First Author	Year	Type	Primary Content
1	*Tafamidis Treatment for Patients with Transthyretin Amyloid Cardiomyopathy*	Mathew S. Maurer	2018	Article	Tafamidis treatment significantly reduced all-cause mortality, cardiovascular-related hospitalizations, and the decline in functional capacity and quality of life in patients with ATTR-CM.
2	*Nonbiopsy Diagnosis of Cardiac Transthyretin Amyloidosis*	Julian D. Gillmore	2016	Article	Bone scintigraphy is a highly sensitive and specific method for diagnosing ATTR-CM without the need for histological confirmation, particularly in patients without monoclonal gammopathy.
3	*Inotersen Treatment for Patients with Hereditary Transthyretin Amyloidosis*	Merrill D. Benson	2018	Article	Inotersen improved neurological function and quality of life in patients with ATTRv amyloidosis, although associated with adverse effects including thrombocytopenia and glomerulonephritis.
4	*Wild-type transthyretin amyloidosis as a cause of heart failure with preserved ejection fraction*	Esther Gonzalez-Lopez	2015	Article	ATTRwt amyloidosis is an underrecognized cause of HFpEF, which can be diagnosed with non-invasive techniques. Specific treatments may improve symptoms and quality of life.
5	*Systemic amyloidosis*	Ashutosh D. Wechalekar	2016	Review	Systemic amyloidosis is a rare condition characterized by the deposition of misfolded proteins in organs, leading to dysfunction, with advancements in diagnosis and treatment improving patient outcomes.
6	*Noninvasive etiologic diagnosis of cardiac amyloidosis using ^{99m}Tc-3,3-diphosphono-1,2-propanodicarboxylic acid scintigraphy*	Enrica Perugini	2005	Article	The effectiveness of ^{99}mTc-DPD scintigraphy as a noninvasive method for diagnosing cardiac amyloidosis, allowing differentiation between ATTR and AL amyloidosis.
7	*Transthyretin Amyloid Cardiomyopathy: State-of-the-Art Review*	Frederick L. Ruberg	2019	Review	The clinical features, diagnostic approaches, and treatment options for ATTR-CM, and the role of early detection and therapies in improving patient outcomes.
8	*Systemic Cardiac Amyloidoses Disease Profiles and Clinical Courses of the 3 Main Types*	Claudio Rapezzi	2009	Article	The distinct clinical courses among AL, ATTRv, and ATTRwt amyloidosis. AL progressing aggressively despite less morphological involvement, while ATTRwt has a slower course despite greater wall thickness.

Table S1 continued

Primary content of the top 10 cited publications

Rank	Publication Title	First Author	Year	Type	Primary Content
9	*Tafamidis, a potent and selective transthyretin kinetic stabilizer that inhibits the amyloid cascade*	Christine E. Bulawa	2012	Article	Tafamidis prevents the aggregation of TTR into amyloid fibrils, offering a therapeutic approach for ATTR amyloidosis.
10	*Diagnosis and treatment of cardiac amyloidosis. A position statement of the European Society of Cardiology Working Group on Myocardial and Pericardial Diseases*	Pablo Garcia-Pavia	2021	Review	The invasive and non-invasive definition of cardiac amyloidosis, clinical scenarios and situations to suspect cardiac amyloidosis, a diagnostic algorithm to aid diagnosis and how to monitor and treat cardiac amyloidosis.

Table S2

Primary content of the top 25 references with the strongest citation bursts

No.	Strength	Reference Title	Type	Primary Content
1	25.22	*Systemic Cardiac Amyloidoses: Disease Profiles and Clinical Courses of the 3 Main Types*	Article	The distinct clinical courses among AL, ATTRv, and ATTRwt amyloidosis. AL progressing aggressively despite less morphological involvement, while ATTRwt has a slower course despite greater wall thickness.
2	24.59	*Transthyretin-related amyloidoses and the heart: a clinical overview*	Review	ATTRv amyloidosis is often underdiagnosed due to its variable symptoms. clinicians should suspect it in patients with unexplained left ventricular wall thickening.
3	21.38	*Role of (^{99}m) Tc-DPD scintigraphy in diagnosis and prognosis of hereditary transthyretin-related cardiac amyloidosis*	Article	(^{99}m) Tc-DPD scintigraphy can identify myocardial infiltration, and is is a prognostic determinant of "cardiac" outcome in ATTR.
4	38.29	*Tafamidis for Transthyretin Familial Amyloid Polyneuropathy: A Randomized, Controlled Trial*	Article	Tafamidis was associated with a trend toward better preservation of neurological function and quality of life in early-stage V30M TTR-FAP patients.
5	22.98	*Disease profile and differential diagnosis of hereditary transthyretin amyloidosis*	Article	Some ATTRv patients are exclusively cardiac involved,mimicing hypertrophic cardiomyopathy and senile ATTRwt amyloidosis.
6	38.14	*Repurposing Diflunisal for Familial Amyloid Polyneuropathy: A Randomized Clinical Trial*	Article	Diflunisal significantly slows the progression of neurological impairment and preserves quality of life in patients with FAP.
7	35.64	*(^{99}m) Tc-pyrophosphate scintigraphy for differentiating light-chain cardiac amyloidosis from the transthyretin-related familial and senile cardiac amyloidoses*	Article	^{99}mTc PYP cardiac imaging effectively differentiates AL from ATTR-CM and presents a simple, widely accessible approach for identifying ATTR-CM.
8	32.65	*Safety and Efficacy of RNAi Therapy for Transthyretin Amyloidosis*	Article	ALN-TTR01 and ALN-TTR02 effectively reduces both mutant and nonmutant transthyretin levels, offering a proof of concept for RNAi therapy.
9	29.33	*Transthyretin (TTR) cardiac amyloidosis*	Review	ATTR-CM is an overlooked cause of heart failure in the elderly. While liver transplantation is the standard for ATTRv, the efficacy of emerging treatments for ATTR gene carriers is needed to be determined.
10	21.72	*Long-term effects of tafamidis for the treatment of transthyretin familial amyloid polyneuropathy*	Article	Long-term tafamidis treatment is well tolerated and effectively slows the progression of neurological impairment in TTR-FAP patients.
11	23.37	*Guideline of transthyretin-related hereditary amyloidosis for clinicians*	Review	Familial amyloidotic polyneuropathy, is a challenging and often misdiagnosed disease, but early recognition and appropriate management can significantly improve outcomes.

Table S2 continued

Primary content of the top 25 references with the strongest citation bursts

No.	Strength	Reference Title	Type	Primary Content
12	22.96	*Left Ventricular Structure and Function in Transthyretin-Related Versus Light-Chain Cardiac Amyloidosis*	Article	Patients with ATTR-CM exhibit greater left ventricular wall thickness and lower ejection fraction compared to those with AL amyloidosis yet have better survival rates.
13	21.68	*Utility and Limitations of 3,3-Diphosphono-1,2-Propanodicarboxylic Acid Scintigraphy in Systemic Amyloidosis*	Article	^{99}mTc-DPD scintigraphy is highly sensitive for imaging ATTR-CM, but it is not specific and must be interpreted within a broad clinical context.
14	44	*Wild-type transthyretin amyloidosis as a cause of heart failure with preserved ejection fraction*	Article	ATTRwt is an underdiagnosed condition, accounting for a significant proportion of HFpEF cases in the elderly population.
15	74.29	*Nonbiopsy Diagnosis of Cardiac Transthyretin Amyloidosis*	Article	Bone scintigraphy is a highly sensitive and specific method for diagnosing ATTR-CM without the need for histological confirmation, particularly in patients without monoclonal gammopathy.
16	28.18	*Diagnosis, Prognosis, and Therapy of Transthyretin Amyloidosis*	Review	The appropriate diagnostic evaluation for those with known or suspected amyloidosis, the currently available interventions.
17	46.14	*Genotype and Phenotype of Transthyretin Cardiac Amyloidosis: THAOS (Transthyretin Amyloidosis Outcomes Survey)*	Article	TTR Val122Ile is the most common mutation in the U.S. patients, and neurologic phenotypic expression differs between wild-type and Val122Ile amyloidosis, but survival does not.
18	28.45	*Inotersen Treatment for Patients with Hereditary Transthyretin Amyloidosis*	Article	Inotersen improved neurological function and quality of life in patients with ATTRv amyloidosis, although associated with adverse effects including thrombocytopenia and glomerulonephritis.
19	28.22	*Patisiran, an RNAi Therapeutic, for Hereditary Transthyretin Amyloidosis*	Article	Patisiran significantly improves the neurological function, quality of life, and mobility of patients with ATTRv amyloidosis with polyneuropathy.
20	24.49	*Unveiling Transthyretin Cardiac Amyloidosis and Its Predictors Among Elderly Patients with Severe Aortic Stenosis Undergoing Transcatheter Aortic Valve Replacement*	Article	ATTR-CM is prevalent among elderly patients with severe calcific aortic stenosis undergoing TAVR, assocиtated with low-flow, low-gradient aortic stenosis with mildly reduced ejection fraction.
21	46.97	*Tafamidis Treatment for Patients with Transthyretin Amyloid Cardiomyopathy*	Article	Tafamidis treatment significantly reduced all-cause mortality, cardiovascular-related hospitalizations, and the decline in functional capacity and quality of life in patients with ATTR-CM.

Table S2 continued

Primary content of the top 25 references with the strongest citation bursts

No.	Strength	Reference Title	Type	Primary Content
22	40.23	*Transthyretin Amyloid Cardiomyopathy: JACC State-of-the-Art Review*	Review	The clinical features, diagnostic approaches, and treatment options for ATTR-CM, and the role of early detection and therapies in improving patient outcomes.
23	21.37	*Expert Consensus Recommendations for the Suspicion and Diagnosis of Transthyretin Cardiac Amyloidosis*	Review	The key clinical indicators and a diagnostic approach involving monoclonal protein testing, scintigraphy, biopsy, and transthyretin genotyping for early recognition of ATTR-CM.
24	29.29	*Cardiac Amyloidosis: Evolving Diagnosis and Management: A Scientific Statement from the American Heart Association*	Review	The importance of early recognition and accurate diagnosis of cardiac amyloidosis, the advancements in noninvasive imaging techniques, and the emerging therapies for ATTR-CM.
25	28.13	*Natural History, Quality of Life, and Outcome in Cardiac Transthyretin Amyloidosis*	Article	There was substantial diagnostic delay in ATTR-CM, and patients experienced substantial impairments in quality of life, especially in those with Val122Ile.

Table S3

Top 25 keywords with the highest co-occurrence frequency

Rank	Keyword	Co-occurrence frequency
1	cardiac amyloidosis	746
2	diagnosis	582
3	heart failure	428
4	polyneuropathy	321
5	transthyretin amyloidosis	288
6	light chain amyloidosis	266
7	liver transplantation	236
8	cardiomyopathy	170
9	familial amyloid polyneuropathy	149
10	systemic amyloidosis	149
11	disease	143
12	variant	139
13	transthyretin amyloid cardiomyopathy	138
14	transthyretin	132
15	cardiovascular magnetic resonance	129
16	senile systemic amyloidosis	122
17	prevalence	121
18	management	119
19	natural history	119
20	echocardiography	107
21	tafamidis	105
22	recommendations	103
23	scintigraphy	101
24	magnetic resonance	97
25	phenotype	89
